# Strategic selection of microbial cell factories for sustainable and application-specific terpenoid production

**DOI:** 10.3389/fbioe.2026.1933337

**Published:** 2026-09-04

**Authors:** Vibha Shukla, Virendra Shukla, Shweta Rawat, Vandana Singh, Suresh Chandra Phulara

**Affiliations:** 1 Independent Researcher, Lucknow, India; 2 Cell Culture Laboratory, Patanjali Food and Herbal Park, Haridwar, Uttarakhand, India; 3 Biotechnology Department, Bipin Tripathi Kumaon Institute of Technology, Dwarahat, Almora, Uttarakhand, India; 4 Department of Biotechnology, Govind Ballabh Pant Institute of Engineering and Technology, Pauri, Uttarakhand, India

**Keywords:** green biomanufacturing, host selection, microbial cell factories, sustainable production, terpenoids

## Abstract

Terpenoids (isoprenoids) constitute one of the largest and most structurally diverse families of natural products with application ranging from flavors, pharmaceuticals to biofuels. Conventional extraction from plants is often limited by low yields, seasonal variability, and environmental constraints; whereas, chemical synthesis requires toxic chemicals and energy-intensive processes. Consequently, microbial cell factories have emerged as sustainable and industrially scalable alternatives for terpenoid biosynthesis. Different microorganisms possess distinct physiological and metabolic advantages, including efficient precursor supply, tolerance to toxic products, internal storage for hydrophobic compounds, utilization of renewable carbon sources, and compatibility with complex biosynthetic pathways. Furthermore, several microbial hosts have strains with Generally Recognized as Safe (GRAS) status, making them attractive candidates for food and nutraceutical applications, although their regulatory acceptance ultimately depends on the production strain, genetic modifications, manufacturing process, product, and intended use. In addition, photosynthetic cyanobacteria offer a promising platform for direct conversion of CO_2_ into terpenoids, providing opportunities for more resource-efficient and sustainable biomanufacturing. Therefore, strategic host selection is a crucial step in designing efficient microbial platforms for terpenoid production. The present review provides a host-centric perspective by comparing conventional and emerging microbial cell factories, highlighting their physiological strengths, product spectrum, industrial applicability, and strategic considerations for sustainable and application-specific terpenoid biomanufacturing.

## 1 Introduction

The terpenoids and terpene-derivatives are structurally and functionally varied classes of natural compounds. They are so vast that they are often termed as “terpenome” and embrace almost one-third of the molecules presently included in the ‘*Dictionary of Natural Products*’ (Hillier and Lathe, 2019). Due to their chemical diversity, terpenoids find diverse applications ranging from flavor (limonene, linalool, geraniol, etc.), pharmaceuticals (artemisinin, taxadiene, ginsenosides, etc.) to advanced biofuel (isopentenols, pinene, farnesene, bisabolene, etc.) (Gupta and Phulara, 2021). Even a single terpenoid molecule can be utilized for several biological and non-biological applications, for example, limonene and limonene derivatives find diverse applications across industries, such as fragrance, flavor, antimicrobial agent, solvent, etc., (Jongedijk et al., 2016).

**FIGURE 1:**
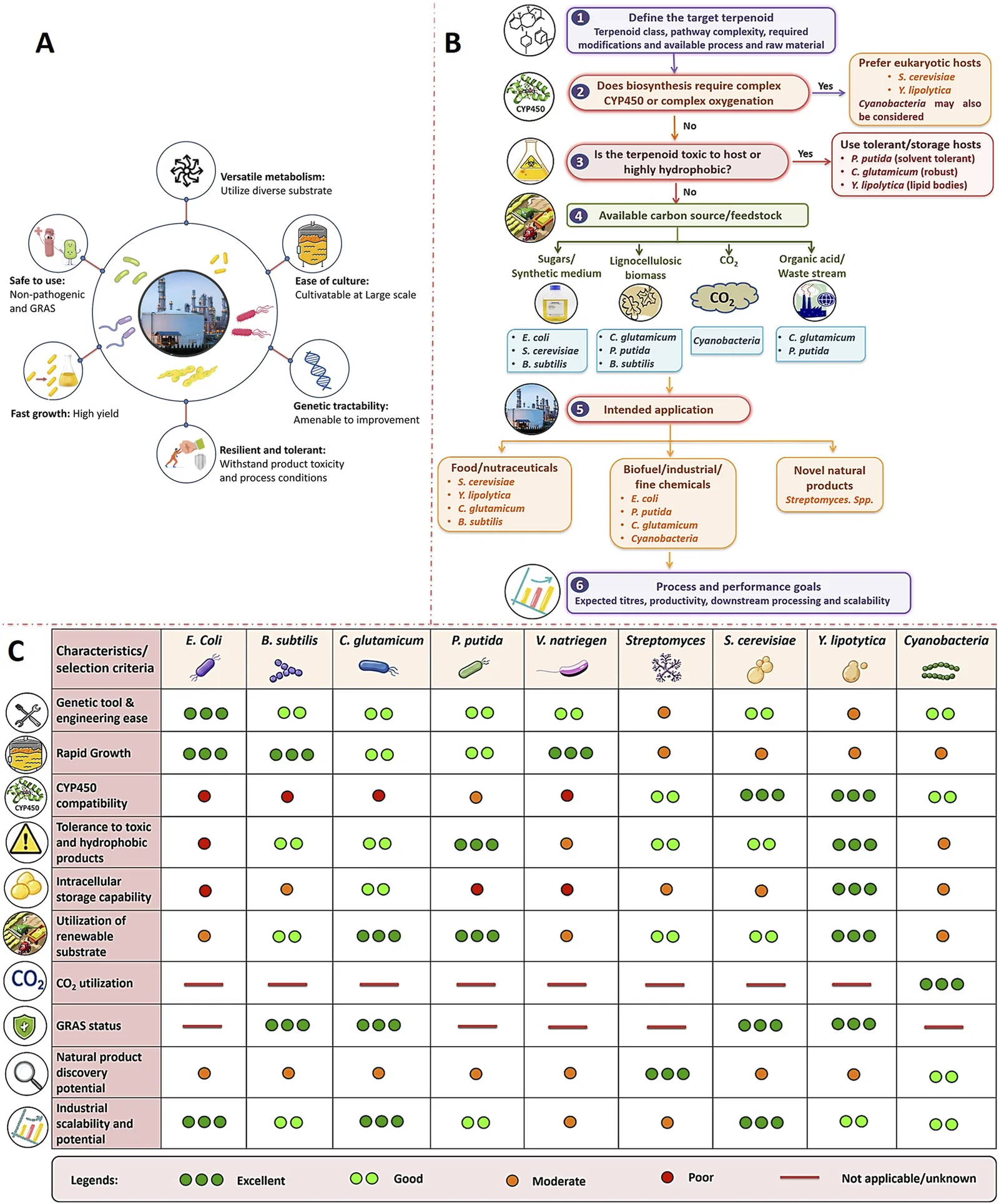
Strategic framework for microbial host selection for sustainable and application-specific terpenoid biomanufacturing. **(A)** Desirable characteristics of an ideal microbial cell factory. **(B)** Decision-making framework for rational microbial host selection. **(C)** Comparative evaluation of conventional and emerging microbial cell factories for terpenoid production. Assessment based on comparative analysis of published literature discussed in this review; ratings represent relative qualitative performance across multiple criteria and are not absolute quantitative rankings.

Currently plant-based extraction is the major source of terpenoids. Chemical synthesis is another route, which is also being utilized currently to meet global demand. However, the limitations in the extraction of terpenoids from plant sources and concerns associated with their chemical synthesis have driven global interest towards the sustainable production of such valuable metabolites from alternative routes (Hu Y. et al., 2025; Zheng et al., 2026). Plant tissue/cell culture techniques have contributed significantly in this regard; however, the cost and technical constraints involved in tissue/cell culture approaches (Kusari et al., 2014) have urged researchers to explore more sustainable approaches for the production of terpenoid-based metabolites. Consequently, microbial cell factories have emerged as attractive alternatives because of their rapid growth, ease of cultivation, amenability to genetic engineering, and compatibility with large-scale fermentation processes (Figure 1A).

Microbial terpenoid biosynthesis occurs mainly through two pathways: the methylerythritol phosphate (MEP) pathway, which is predominant in most bacteria and cyanobacteria, and the mevalonate (MVA) pathway, which is naturally present in fungi and a few bacteria. Both the pathways generate the universal precursors, isopentenyl pyrophosphate (IPP) and dimethylallyl pyrophosphate (DMAPP), for downstream terpenoid biosynthesis (Gupta and Phulara, 2021). Over the past two decades, rapid advances in metabolic engineering, synthetic biology, genome editing and systems biology have remarkably improved the capability of microorganisms to produce a wide range of industrially relevant terpenoids (Pabbathi et al., 2025; Zheng et al., 2026) (Table 1). Although, *Escherichia coli* and *Saccharomyces cerevisiae* remain the most extensively used microbial platforms for terpenoid biosynthesis; however, recent years have witnessed growing interest in several alternative microbial hosts due to their distinct physiological and metabolic advantages, such as higher precursor availability, compatibility with cytochrome P450-dependent reactions, tolerance to toxic terpenoids, utilization of renewable feedstocks, and regulatory (Pramastya et al., 2021; Hu et al., 2023; Luckie et al., 2024; Zhong et al., 2024; Liu et al., 2026).

**TABLE 1:** Representative microbial production of terpenoids across different classes, production hosts, fermentation strategies, and achieved titers.

Terpenoid class	Terpenoid	Primary use/Function	Production host	Reported production metric*	Fermentation mode/Carbon source	References
Hemiterpene	Isoprene	Industrial precursor	*E. coli*	60 g/L	Fed batch	(Navale et al., 2021)
			*S. cerevisiae*	2.5 g/L	Fed batch (Sucrose)	
			*B. subtilis*	1.4 mg/L	Shake flask	(Pramastya et al., 2021)
	Isopentenol	Gasoline additive, nutraceutical, cosmetic, flavour, fragrance	*E. coli*	8.51 g/L	Fed batch (Glucose)	(Wang et al., 2022a)
			*C. glutamicum*	1.25 g/L	Shake flask (Glucose)	(Sasaki et al., 2019)
				1.1 g/L	Shake flask (Ionic liquid treated biomass hydrolysate)	
			*V. natriegens*	2.76 mg/L	Shake flask (crude kelp powder)	(Lee et al., 2024)
			*P. putida*	3.5 g/L	Fed-batch (Glucose)	(Banerjee et al., 2024)
				0.4 g/L	Shake flask (Biomass hydrolysate)	
			*S. cerevisiae*	1.57 g/L	24-well microtiter plates (Glucose)	(Xiao et al., 2025)
			*B. subtilis*	6.8 mg/L	Shake flask (Glucose)	(Phulara et al., 2018a)
				2.6 mg/L	Shake flask (Biomass hydrolysate)	
			*Cyanobacteria*	105.2 mg/L	continuous light conditions	(Zhou et al., 2024)
Monoterpene	Pinene	Jet fuel precursor, flavouring, fragrance	*E. coli*	0.97 g/L	Fed-batch (Glucose)	(Navale et al., 2021)
			*S. cerevisiae*	1.8 g/L	Fed-batch (Glucose)	(Zhang et al., 2025)
			*Y. lipolytica*	36.1 mg/L	Ligocellulosic hyrolysate	(Wei et al., 2021)
				33.6 mg/L	Waste cooking oil	
			*Cyanobacteria*	1.52 mg/L	continuous light conditions	(Yang et al., 2021)☺
	Geranic acid	Prefume, flavour	*P. putida*	139 mg/L	Fed-batch (Glycerol)	(Mi et al., 2014)
	Limonene	Prefume, flavour, jet fuel precursor	*S. cerevisiae*	15.2 g/L	Fed-batch (Glycerol)	(Ma et al., 2026)
			*E. coli*	3.6 g/L	Fed-batch (Glycerol)	(Rolf et al., 2020)
			*Y. lipolytica*	91.24 mg/L	Shake flask (Waste cooking oil)	(Li et al., 2022)
			*Cyanobacteria*	1.7 mg/L	continuous light conditions	(Chenebault et al., 2023)
Sesquiterpene	Farnesene	Lubricants, polymers, Diesel substitute	*E. coli*	62.29 g/L	Fed-batch (Glucose)	(Men et al., 2026)
				10.31 g/L	Fed-batch (Crude glycerol)	(Yao et al., 2020)
			*Y. lipolytica*	82.1 g/L	Fed-batch (glucose and oleic acid)	(Liu et al., 2026)
​	*​*	*​*	*S. cerevisiae*	10.4 g/L	Fed-batch (Glucose)	(Wang et al., 2021)
			*Cyanobacteria*	30 mg/L	continuous light conditions	(Blanc-Garin et al., 2022)
	Amorphadiene	Pharmaceutical (precursor to anti-malarial drug artemisinin)	*Y. lipolytica*	65.094 mg/L	Shake flask (Glucose)	(Marsafari et al., 2022)
			*B. subtilis*	116 mg/L	Shake flask	(Song et al., 2021)
			*S. cerevisiae*	40 g/L	Fed-batch (Glucose)	(Westfall et al., 2012)
			*E. coli*	30 g/L	Fed-batch (Glucose)	(Shukal et al., 2019)
	Pentalenene	Pharmaceutical, jet fuel precursor	*V. natriegens*	39.4 mg/L	Shake flask (Glucose)	(Hu et al., 2025a)
Diterpene	Taxadiene	Pharmaceutical (precursor to anti-cancer drug Taxol)	*E. coli*	1 g/L	Fed-batch (Glucose)	(Ajikumar et al., 2010)
			*B. subtilis*	17.8 mg/L	Shake flask (Glucose)	(Abdallah et al., 2019)
			*S. cerevisiae*	1.26 g/L	Fed-batch (Glucose)	(Zhang et al., 2026)
	Taxadiene-5α-ol	Pharmaceutical (precursor to anti-cancer drug Taxol)	*cyanobacteria*	4.32 mg/L	continuous light conditions	(Zhong et al., 2024)
	Ratinol	Pharmaceutical, nutraceutical	*S. cerevisiae*	7.19 g/L	Fed-batch	(Song et al., 2026)
Triterpene	Squalene	Pharmaceutical, cosmetic	*S. cerevisiae*	55.8 g/L	Fed-batch (Glucose)	(Tang et al., 2025)
			*Y. lipolytica*	4.53 g/L	Fed-batch (Glucose)	(Zhao et al., 2026)
			*C. glutamicum*	82.8 mg/L	Shake flask (Glucose)	(Park et al., 2019)
			*B. subtilis*	7.5 mg/L	Shake flask	(Song et al., 2020)
Caratenoids	decaprenoxanthin	Food, cosmetic, nutraceutical	*C. glutamicum*	1.45 g/L	Fed-batch (Glucose)	(Stegelmann et al., 2026)
				129.6 mg/L	Biomass hydrolysate	
	α-carotene		*C. glutamicum*	1.8 g/L	Fed-batch (Glucose and xylose)	(Li et al., 2024)
				216 mg/L	Shake flask (Biomass hydrolysate)	
	β-carotene		*Y. lipolytica*	6.5 g/L	Fed-batch (Glucose)	(Larroude et al., 2018)
			*Cyanobacteria*	4.03 mg/g dry weight	continuous light conditions	(Pagels et al., 2021)
	Lycopene		*Y. lipolytica*	4.2 g/L	Shake flask (Glucose)	(Luo et al., 2020)

* Production values are presented as either volumetric titres (g/L or mg/L) or intracellular product content (mg/g dry cell weight), as reported in the original studies. Since the studies used different hosts, engineering strategies, carbon sources, and cultivation modes, the reported values represent indicative production capabilities and should not be interpreted as directly comparable performance metrics.

There are several excellent reviews available in public domains, which have comprehensively discussed metabolic engineering and synthetic biology strategies for terpenoid production (Hu Y. et al., 2025; Pabbathi et al., 2025; Wang et al., 2025; Zheng et al., 2026). To the best of our knowledge, comparatively less attention has been given to the rational selection of microbial cell factories according to the biochemical complexity of terpenoids, intended industrial application, regulatory requirements, and process sustainability. In this context, the present review provides a host-centric perspective on conventional and emerging microbial cell factories for terpenoid production. It highlights the inherent strengths of individual hosts and presents a practical framework for selecting suitable microbial chassis for sustainable, green and application-specific terpenoid biomanufacturing.

## 2 Microbial hosts for terpenoid production

Despite the remarkable progress in metabolic engineering and synthetic biology, no single microorganism is ideally suited for the production of all classes of terpenoids. The physiological and metabolic diversity among microbial hosts largely determines precursor availability, tolerance to toxic products, substrate utilization, scalability, and regulatory acceptance (Figure 1A). Therefore, selecting an appropriate microbial chassis is a key step toward efficient and sustainable terpenoid biomanufacturing. In this context, the following subsections discusses the physiological and metabolic attributes of conventional and emerging microbial cell factories along with the recent advances to facilitate strategic host selection for green and application-specific terpenoid production.

### 2.1 *Escherichia coli,* an ideal host for validating novel genes and pathways

*Escherichia coli* is the most widely utilized microbial chassis for the production of various primary and secondary metabolites, including terpenoids (Hu Y. et al., 2025; Pabbathi et al., 2025). This is due to the ease in its culturing, handling, and superiority at cloning and expressing foreign proteins. The rapid growth rate of *E. coli* in chemically defined medium supports the growth of high-density cultures even in recombinant form. Due to this, *E. coli* often dominates in microbial fermentations and its output is high relative to other microbial sources (Pabbathi et al., 2025; Zheng et al., 2026). Moreover, in the majority of the cases, *E. coli* is used for genetic manipulations of shuttle vectors before transforming them into alternate bacterial hosts, like *Bacillus subtilis*, *Corynebacterium glutamicum*, etc. (Zhou et al., 2013; Kang et al., 2014; Phulara et al., 2018b).

*Escherichia coli* has not only served as a microbial platform for terpenoid production, but also has helped in improving our understanding in the area of microbial terpenoid production. For example, entire heterologous MVA pathway was first introduced and expressed in *E. coli* (Martin et al., 2003). Since then, it was the most widely utilized strategy for the non-natural terpenoids production of terpenoids (Phulara et al., 2016; Zheng et al., 2026) until an isopentenol utilization (IU) (Chatzivasileiou et al., 2019) was not demonstrated in *E. coli*. MVA-mediated IPP-Bypass (MMIB) (Kang et al., 2016) and Repass (MMIR) (Carruthers et al., 2025a) pathways have also been demonstrated in *E. coli* first. Later, these strategies were successfully utilized in alternate microbial chassis, such as *C. glutamicum* (Sasaki et al., 2019) and yeasts (Ge et al., 2025; Garcia et al., 2026). Similarly, several other discoveries in *E. coli* also contributed to enhance the knowledge base in the field of microbial terpenoid production, such as role of NudX (Withers et al., 2007) hydrolases in rescuing microbial hosts from prenyl precursors (IPP and DMAPP) induced toxicity, biosynthesis of IPP and DMAPP in a ratio 5:1 (Rohdich et al., 2002), insoluble nature of DXP pathway enzymes (Zhou et al., 2012a), efflux of methylerythritol cyclodiphosphate (MEcPP) (Zhou et al., 2012b) and feedback inhibition of DXP synthase (DXS) by IPP and DMAPP (Banerjee et al., 2013). In addition to this, the harnessing of efflux pumps can alleviate product-induced toxicity and improve terpenoid titers in microbial hosts were also demonstrated in *E. coli* (Foo and Leong, 2013).

Utilizing *E. coli* as model chassis, a novel route to DXP synthesis has also been designed that can biosynthesize DXP directly from C5 sugars (Kirby et al., 2015). Currently, scientific communities across the globe are exploring CRISPR interference (CRISPRi) in *E. coli* for fine-tuning metabolic networks to improve terpenoid titers in microbial hosts (Wang J. et al., 2022; Kim and Lee, 2023). Utilizing CRISPRi in *E. coli*, recent studies have demonstrated that multiplex guide RNA strategy instead single-gene repression is more beneficial in redirecting carbon flux away from competing metabolic pathways (Phulara et al., 2026). Thus, the contribution of *E. coli* in the progress of microbial terpenoid production is ahead of other alternate microbial hosts.

### 2.2 *Saccharomyces cerevisiae*, a promising host for structurally complex terpenoid biosynthesis

In eukaryotic system, *S*. *cerevisiae* has established itself as an attractive chassis for *de-novo* terpenoid synthesis, due to its well-characterized MVA pathway, robustness for genetic modulation and compatibility for industrial bioprocessing (Ma et al., 2023). In addition to its GRAS status, engineering strategies for efficient and functional expression of cytochrome P450 enzymes (CYPs) for further terpenoid scaffold decoration bring it to the forefront of terpenoid based flavors and pharmaceuticals. The production of structurally complex terpenoid molecules like artemisinin or paclitaxel requires multiple oxidation and functionalization steps. These reactions are largely governed by CYPs, which, together with terpene synthases, dictate the regio- and stereochemical diversity of bioactive terpenoids (Shukla et al., 2023). Thus, functional expression of CYPs is crucial for expanding the chemical complexity and functional value of terpenoid products in microbial systems.

Yeasts, such as *S. cerevisiae*, provide a suitable intracellular environment for CYPs activity due to its endoplasmic reticulum membrane system, which supports proper enzyme localization and folding, along with a relatively low background of competing endogenous CYPs (Bureau et al., 2023). However, overexpression of CYPs may impose metabolic stress, particularly due to increased demand for heme, which is a critical cofactor required for oxygen activation and catalytic turnover. In addition, efficient functioning of CYPs depends on their interaction with cytochrome P450 reductase (CPR), which transfers electrons from NADPH to the hemecenter of CYPs to drive catalytic activity. The compatibility between heterologous CYPs and CPR significantly influences overall pathway efficiency (Wang et al., 2025). Therefore, co-expression of an appropriate CPR is essential for optimal performance. Strategies such as enhancing heme biosynthesis by overexpressing rate-limiting genes like *HEM13* have been successfully employed in *S. cerevisiae* to improve CYP activity and alleviate cellular burden, leading to enhanced terpenoid production (Wang et al., 2025).

Overall, *S. cerevisiae* offers a robust and versatile platform for CYP-mediated terpenoid biosynthesis that is challenging in prokaryotic hosts like *E. coli*. However, the success relies on balancing of redox partners, cofactor availability, and metabolic flux. Continued and careful optimization of these factors would be key to unlocking the full potential of yeast as a green cell factory for high-value and structurally complex terpenoids for food and pharmaceutical applications.

### 2.3 Hosts for flavor, pharmaceuticals and nutraceuticals

Although, *E. coli* is preferred for rapid pathway engineering because of its fast growth and robust genetic toolbox and *S. cerevisiae* is suitable for structurally complex, cytochrome P450-dependent terpenoids. However, limitations such as endotoxin production in *E. coli* (Mamat et al., 2015) and the relatively slower growth and complex cellular organization of *S. cerevisiae* (Paramasivan and Mutturi, 2017) have driven global interest for exploring alternative GRAS microbial hosts with distinct industrial advantages. Microbial hosts, such as *B. subtilis, C. glutamicum*, and *Yarrowia lipolytica*, discussed below, have shown considerable potential in this regard. However, regulatory acceptance while utilizing these hosts depends on the production strain, genetic modifications, intended use, manufacturing process, purification strategy, and control of residual impurities.

#### 2.3.1 Bacillus subtilis

*Bacillus subtilis*, a Gram-positive, endospore-forming bacterium belonging to the order *Bacillales*, has emerged as one of the promising GRAS microbial platforms for terpenoid biosynthesis. Comparative genomic analyses have further revealed that *Bacillales* harbor a larger repertoire of genes related to terpenoid biosynthesis than many other bacterial groups (Guan et al., 2015). Notably, *B. subtilis* has been reported to produce nearly 18-fold higher levels of isoprene than *E. coli* (Fall and Copley, 2000). This indicates that a relatively abundant intracellular pool of the universal terpenoid precursors, IPP and DMAPP might be present in *Bacillae,* including *B. subtilis*. Together with its rapid doubling time (∼30 min), and extensive genetic toolbox, *B. subtilis* becomes an attractive chassis for sustainable terpenoid production.

This microbial cell factory can efficiently metabolize both hexose and pentose sugars derived from minimally pretreated lignocellulosic biomass (Phulara et al., 2018b); therefore, reducing the need for extensive pretreatment of biomass or additional pathway engineering. Its natural cellulolytic capability enables it to partially decompose lignocellulosic biomass without supplementing costly hydrolytic enzymes (Hohmann et al., 2016). Another distinctive advantage is its ability to form highly resistant endospores under unfavorable conditions, which allows long-term storage, easy transportation, and rapid regeneration of vegetative cells. However, sporulation may also complicate large-scale biomanufacturing by introducing process heterogeneity and increasing contamination-control and downstream processing requirements. Therefore, appropriate assessment of residual cells, host-derived components, and other process-related impurities is required according to the intended use and applicable regulatory requirements.

Although terpenoid engineering in *B. subtilis* remains less explored than in *E. coli* or *S. cerevisiae*, significant progress has been achieved in recent years. Natural and non-natural production of diverse terpenoid classes, ranging from hemiterpenoids to carotenoids, has been demonstrated through metabolic engineering (Pramastya et al., 2021). Majority of the engineering efforts in *B. subtilis* have focused on modulating endogenous MEP pathway by overexpressing native pathway enzymes together with product-specific synthases, (Phulara et al., 2018b; Abdallah et al., 2019; Song et al., 2020; Yang et al., 2020), thereby overcoming the comparatively lower efficiency of expressing multiple heterologous genes. Successful establishment of CRISPR-Cas9 system in *B. subtilis* has opened the new gateway for achieving enhanced titers of therapeutic terpenoids, such as amorphadiene (Song et al., 2021). Further, more dedicated studies on fine-tuning metabolic networks using CRISPRi might improve terpenoid titers several folds in this promising microbial chassis by redirecting carbon flux away from metabolic pathways that compete for precursors from MEP pathway.

#### 2.3.2 Corynebacterium glutamicum

*Corynebacterium glutamicum* is one of the most established industrial microorganisms and has been extensively used for the safe, large-scale production of amino acids for more than five decades (Becker and Wittmann, 2016; Henke et al., 2018b). The potential of *C. glutamicum* for terpenoid production was first demonstrated through the successful biosynthesis of lycopene (Heider et al., 2012) and was subsequently expanded to several other commercially important carotenoids, including α- and β-carotene, zeaxanthin, canthaxanthin, astaxanthin, and decaprenoxanthin (Heider et al., 2014b; 2014a; Henke et al., 2016; 2018a; Li et al., 2021; Stegelmann et al., 2026). In parallel, terpenoids beyond carotenoids, such as hemiterpenoids, monoterpenes, and sesquiterpenes have also been produced through this microbial (Sasaki et al., 2019; Luckie et al., 2024; Atas Erden et al., 2025). Most importantly, unlike *B. subtilis,* studies on *C. glutamicum* explored MVA, MVA dependent-IPP bypass and MEP-mediated modulations to achieve non-natural production of diverse class of (Henke et al., 2018b; Sasaki et al., 2019; Luckie et al., 2024). These achievements clearly demonstrate the versatility of *C. glutamicum* as a production host for diverse classes of terpenoids.

Recent studies have further strengthened its production capacity by engineering its native carotenoid pathway along with genome editing and pathway optimization. This enabled the engineered strain to produce 1.45 g/L of decaprenoxanthin in fed-batch fermentation, which represents the highest C50 carotenoid titer reported to date (Stegelmann et al., 2026). Through systematic optimization of plasmid architecture, promoter strength, and geranyl diphosphate synthase combined with elimination of competing pathways and a biphasic cultivation strategy, the engineered *C. glutamicum* strain produced record levels of isoprenol 1.50 g/L (Luckie et al., 2024) amongst all alternate microbes utilized till date. This platform was also successfully utilized for the production of monoterpenes, such as geranoids, 1,8-cineole, and linalool. The study also uncovered previously unknown monoterpene metabolism in *C. glutamicum*, including the role of the aldehyde reductase, AdhC, in converting acyclic monoterpene aldehydes (Luckie et al., 2024). Optimization of carbon utilization through metabolic rewiring in *C. glutamicum* has shown to enable efficient co-utilization of sugars, such as glucose, xylose, and acetate for α-carotene biosynthesis. By disconnecting glycolysis from the TCA cycle and redirecting carbon flux toward the isoprenoid pathway, the engineered strain achieved 1.80 g/L α-carotene in fed-batch fermentation, which is the highest microbial titer reported to date (Li et al., 2024). These findings may further expand the metabolic engineering toolbox for monoterpene and carotenoids production in other microbial chassis also.

Like *B. subtilis, C. glutamicum* can metabolize renewable feedstocks such as glycerol, xylose, and arabinose efficiently for terpenoid production (Atas Erden et al., 2025), highlighting its potential for sustainable terpenoid production. In addition, this microbial strain has also been found capable of producing terpenoids by utilizing ionic liquid pre-treated plant biomass, which generally inhibits the growth of many host strains (Sasaki et al., 2019). Together, these findings indicate that this microbial chassis could be an excellent alternative for addressing the constraints associated with lignocellulosic hydrolysates.

#### 2.3.3 Yarrowia lipolytica

Among alternate yeast models, *Y. lipolytica* has emerged as one of the most promising non-conventional hosts for safe terpenoid biosynthesis owing to its oleaginous nature, GRAS status, and remarkable metabolic flexibility. Unlike *S*. *cerevisiae*, *Y. lipolytica* possesses an inherently high acetyl-CoA flux, making it suitable for the biosynthesis of acetyl-CoA-derived compounds such as terpenoids and polyketides (Liu et al., 2019). The yeast is able to efficiently utilize hydrophobic substrates like lipids, glycerol, and several other low-cost renewable feedstocks and can tolerate a broad range of pH and salinity. Advancement in genome engineering and synthetic biology tools together with these physiological characteristics have established *Y. lipolytica* as a versatile platform for industrial terpenoid production (Liu et al., 2019).

A unique advantage of choosing *Y. lipolytica* is the close metabolic relationship between lipid and terpenoid biosynthesis, as both the pathways share acetyl-CoA as a common precursor. In addition, the organism naturally synthesizes geranylgeranyl diphosphate (GGPP), providing a strong metabolic foundation for higher order terpenoids production, such as carotenoids (Larroude et al., 2018). Moreover, the lipid-rich intracellular environment of *Y. lipolytica* facilitates sequestration of hydrophobic terpenoids, thereby reducing product toxicity and improving intracellular terpenoid accumulation (Larroude et al., 2018; Liu et al., 2019; Zhang et al., 2020). This advantage can be further enhanced by engineering membrane lipid composition, which has been shown to improve terpenoid secretion and has enabled record production of lupeol and other triterpenes and sesquiterpenes (Zhang et al., 2020). Importantly, enhanced lipid accumulation in this host organism has been correlated with improved titers of terpenoids, such as lycopene (titers exceeding 4 g/L) and β-carotene (production of 6.5 g/L), in fed-batch fermentation (Larroude et al., 2018; Luo et al., 2020).

Recent advances in metabolic engineering have further positioned *Y. lipolytica* as a high-performance terpenoid cell factory. Comprehensive metabolic rewiring eliminated carbon overflow metabolism and redirected carbon flux toward acetyl-CoAin *Y. lipolytica*. This enabled β-farnesene production at a level 82.1 g/L, which is the highest terpenoid titer reported in *Y. lipolytica* to date (Liu et al., 2026). Together, the studies indicate that, beyond conventional pathway engineering, the intrinsic lipid metabolism of *Y. lipolytica* might be a strategic target to improve both terpenoid synthesis and product export.

### 2.4 Other microbial communities

Microbial communities, such as *Vibrio natriegens*, *Pseudomonas* sp. and *Streptomyces* sp. have also been explored for sustainable production of terpenoid-based metabolites. If the genetic tractability and GRAS status can be overlooked to choose a host microbe, these microorganisms can be an interesting chassis for the biotechnological production of terpenoids. This is due to their fast growth, ability to withstand harsh conditions and toxic chemicals, and wide array of substrate range.

#### 2.4.1 *Vibrio natriegens:* a fast growing host for metabolic engineering

*Vibrio natriegens* has recently emerged as a next-generation microbial chassis for metabolic engineering due to its exceptionally rapid growth, high substrate uptake rates, and metabolic versatility (Lee et al., 2024). With a doubling time of less than 10 min, it grows considerably faster than conventional hosts such as *E. coli*, which allows rapid strain development and fermentation processes. It is compatible with widely used pET expression systems, supports efficient heterologous protein expression, and possesses a growing synthetic biology toolbox, including CRISPRi (Xu et al., 2022). Together these, its ability to efficiently metabolize a diverse range of carbon sources, including glucose, sucrose, glycerol, arabinose, and galactose, makes it an attractive platform for industrial bio-manufacturing (Xu et al., 2022; Lee et al., 2024).

In the context of terpenoid biosynthesis, *V. natriegens* is still an emerging but highly promising host. Heterologous expression of the MVA pathway has enabled production of terpenoids, such as isopentenol and pentalenene in this microbial host, while pathway optimization significantly improved product titers and substrate utilization (Lee et al., 2024; Hu L. et al., 2025). Transcriptomic analyses revealed that introduction of the MVA pathway led to limited availability of ATP, NADPH, and acetyl-CoA, together with the inactivation or downregulation of the native MEP pathway in this microbial host (Hu L. et al., 2025). Consequently, the efficiency of terpenoid production remained constrained, despite significant upregulation of key MVA pathway genes. In addition, unlike other *Vibrio* species, which thrive in marine environments, *V. Natriegens* lacks components necessary for alginate assimilation (Lee et al., 2024). Together, these constraints restrict precursor supply and overall pathway efficiency in this microbial chassis. Metabolic rewiring, in this regard, has shown to address certain constraints. For example, introduction of an alginate utilization pathway enabled *V. natriegens* to utilize unconventional feedstocks such as marine biomass (Lee et al., 2024), demonstrating its potential for sustainable marine biomass valorization.

Recent advances in genetic tool development, particularly the discovery of TfoX-mediated natural competence, have transformed this once genetically intractable bacterium into a programmable host capable of efficient natural transformation, iterative genome engineering, and scalable strain development (Zhong et al., 2026). With the advancement of metabolic engineering tools, pathway optimization is expected to accelerate for this microbial host.

#### 2.4.2 Pseudomonas putida

Owing to its exceptional metabolic versatility, high redox capacity, and remarkable tolerance to toxic compounds, *Pseudomonas putida* has emerged as a robust non-conventional bacterial host for terpenoid biosynthesis (Poblete-Castro et al., 2017). Unlike many traditional microbial hosts, *P. putida* can thrive under harsh cultivation conditions, requires relatively simple nutrients, and efficiently metabolizes a broad spectrum of renewable carbon sources, such as lignocellulosic sugars, aromatic compounds, organic acids, and plant-derived phenolics (Molina et al., 2014; Loeschcke and Thies, 2015). Furthermore, its membrane remodeling ability and efficient efflux systems, which make it natural solvent tolerant, help it to withstand high intracellular concentrations of terpenoids that are often toxic to *E. coli* and other microbial hosts (Mi et al., 2014).

The terpenoid-producing capability of *P. putida* was initially demonstrated through the heterologous production of the carotenoid zeaxanthin, which also recognized its compatibility with foreign terpenoid biosynthetic pathways (Beuttler et al., 2011). Since then, its product spectrum has been expanded to several other terpenoids, such as isoprenol (hemiterpene), geranic acid (monoterpene), and epi-isozizaene (sesquiterpene) (Mi et al., 2014; Wang X. et al., 2022). Besides *de-novo* biosynthesis, *P. putida* also possesses excellent biotransformation capabilities. This allows it to convert naturally occurring terpenoids into high-value flavoring agents, pharmaceuticals, and anticancer compounds (Loeschcke and Thies, 2015). The dual capability of terpenoid biosynthesis and bioconversion makes *P. putida* a unique platform for producing compounds, which are difficult or costly to obtain via plant extraction or chemical synthesis.

Recent advancements have further strengthened the industrial relevance of *P. putida* for sustainable terpenoid production. The organism has been successfully engineered to produce terpenoid-based metabolites directly from renewable feedstocks including lignocellulosic sugars, aromatic compounds, acetate, and mixed carbon streams, demonstrating its suitability for biomass-based biorefineries (Wang X. et al., 2022; Banerjee et al., 2024; de Siqueira et al., 2025). Further, integration of computational modeling, laboratory automation, CRISPRi, and machine learning has accelerated and strengthened strain optimization, resulting in considerable improvements in terpenoid titers from *P. putida* (Carruthers et al., 2025b), thus demonstrating the power of data-driven metabolic engineering in this host. Together, the combination of exceptional solvent tolerance, metabolic flexibility, renewable feedstock utilization, and rapidly advancing synthetic biology tools establishes *P. putida* as one of the most promising bacterial platforms for the sustainable production of terpenoids, particularly for biofuels, flavors, fragrances, and high-value specialty chemicals.

#### 2.4.3 *Streptomyces* spp.

*Streptomyces* species are well known as prolific producers of structurally and functionally diverse secondary metabolites, such as antibiotics, anticancer agents, immunosuppressants, and terpenoids. Thanks to their diverse post-modification machinery, which allows glycosylation, hydroxylation, phosphorylation, acetylation, and prenylation of biological molecules including terpenoids, thereby generating structurally complex molecules with diverse biological activities (Liu et al., 2018; Nofiani et al., 2026). Importantly, unlike most bacterial communities that rely solely on the MEP pathway, several *Streptomyces* species possess both the MEP and MVA pathways. It provides greater metabolic flexibility and precursor availability for terpenoid biosynthesis via this microbial chassis (Khalid et al., 2017).

Beyond being natural producers, *Streptomyces* have emerged as valuable chassis for terpenoid discovery and biosynthesis. Their compatibility with bacterial terpene synthases and post-modification enzymes, particularly cytochrome P450s, has enabled the functional characterization of previously uncharacterized, genome-mined terpenoid biosynthetic gene clusters. Genome mining coupled with heterologous expression in *Streptomyces* has led to the discovery of several new terpene backbones and many novel terpenoids (Hu et al., 2023). Similarly, recent genome-guided investigations have uncovered several previously unknown terpene synthases capable of producing novel sesquiterpenes, diterpenes, and sesterterpenes (Li et al., 2025). Recently, it was found that modulations in cultivation strategies, such as addition of chemical elicitors, could activate dormant biosynthetic pathways, leading to the production of antimicrobial, cytotoxic, flavor, and fragrance terpenoids (Nofiani et al., 2026). Metabolomic analyses of these terpenoids revealed that several *Streptomyces* metabolites remain chemically uncharacterized. Together these merits highlight the utility of this host for expanding bacterial terpenoid diversity and also indicate that *Streptomyces* remains an unexplored reservoir of unique terpenoid biocatalysts and natural products.

### 2.5 Cyanobacteria: photosynthetic platform for green terpenoid biomanufacturing

Cyanobacteria have emerged as attractive microbial platforms for green terpenoid biomanufacturing because they directly convert atmospheric CO_2_ and solar energy into valuable products through oxygenic photosynthesis. Unlike conventional heterotrophic hosts that depend on organic carbon sources such as glucose or glycerol, cyanobacteria can produce terpenoids using light, water, and CO_2_, making them potentially attractive platform for carbon efficient terpenoid production (Feng et al., 2026). However, the overall environmental sustainability depends on cultivation conditions, which include lighting, mixing, aeration, harvesting, and downstream processing. Model strains such as *Synechocystis* sp. PCC 6803 and *Synechococcus elongatus* PCC 7942, PCC 11901, and its fast-growing strain UTEX 2973 have been extensively engineered for terpenoid production (Feng et al., 2026).

Another important advantage of cyanobacteria is their close connection between photosynthesis and terpenoid biosynthesis. Their abundant thylakoid membranes provide suitable sites for membrane-associated enzymes, including cytochrome P450s. Whereas, the photosynthetic electron transport chain provides a regular reducing power supply through the ferredoxin–ferredoxin-NADP^+^ reductase system, which can support P450-catalyzed oxidation reactions (Zheng et al., 2022). Genome-wide studies have further revealed a rich diversity of P450s and secondary metabolite biosynthetic gene clusters in cyanobacteria (Khumalo et al., 2020). Furthermore, photosynthetic production of the oxygenated taxol intermediate, taxadiene-5α-ol, confirmed the ability of cyanobacteria to support complex cytochrome P450-dependent terpenoid biosynthesis (Zhong et al., 2024). Together, these findings highlight their unexplored potential and position them as promising bio-solar cell factories for next-generation industrially relevant terpenoids.

Recent studies have demonstrated the production of diverse terpenoids in cyanobacteria, including isoprene, isopentenol, pinene, bisabolene, and oxygenated taxanes (Rodrigues et al., 2023; Zhou et al., 2024). A notable breakthrough was the development of the Ru5P shunt, which bypasses the tightly regulated DXS-catalyzed step and directly channels ribulose-5-phosphate from the Calvin–Benson cycle into the MEP pathway. This strategy not only improved carbon utilization from CO_2_ in cyanobacteria, but also resulted in enhanced terpenoid titers, which are amongst the highest reported photosynthetic yield (Zhou et al., 2024). Although, current titers remain lower than those of established heterotrophic hosts; however, rapid advancements in photosynthetic engineering, carbon-fixation pathways, and synthetic biology are steadily improving productivity and expanding the industrial relevance of cyanobacterial terpenoid platforms.

## 3 Strategic framework for host selection

Although, *E. coli* remains the preferred chassis for rapid pathway construction and proof-of-concept studies due to its extensive genetic toolbox and ease of engineering. However, rapid expansion of microbial cell factories and terpenoid-based research on them has revealed that no single microbial chassis is universally suitable for biomanufacturing every class of terpenoid from every available raw material and process conditions. Instead, selection of microbial hosts is a crucial step for successful strain development for desired terpenoid production. This includes matching intrinsic physiological and metabolic characteristics of the host with the target molecules, available raw material and intended industrial application. Therefore, microbial host selection should not only consider maximizing production titer but also incorporate multiple biological, engineering, and regulatory considerations.

One of the considerations is the structural complexity of the target terpenoid. Simple terpenoids, such as hemiterpene, monoterpenes and few sesquiterpenes can generally be synthesized efficiently in bacterial hosts; whereas oxygenated sesquiterpenes, diterpenoids and triterpenoids that require multiple cytochrome P450-mediated oxidation steps are better fit for eukaryotic hosts possessing endoplasmic-reticulum-associated CYP450 systems, particularly *S. cerevisiae* and *Y. lipolytica* (Bureau et al., 2023; Wang et al., 2025). However, while considering these hosts native precursor metabolism should also be considered, as MVA pathway hosts differ in carbon efficiency, precursor availability, and engineering strategies than the MEP pathway hosts.

Product characteristics also play an important role during host selection. For the production of hydrophobic or toxic terpenoids, microorganisms possessing strong solvent tolerance, efficient membrane adaptation, intracellular storage capacity, or active efflux mechanisms are generally preferred. In this regard, *P. putida* emerges as an advantageous prokaryotic chassis because of its exceptional solvent tolerance, whereas the lipid-rich intracellular environment of the yeast *Y. lipolytica* efficiently sequesters lipophilic terpenoids and places it at the forefront for the production of hydrophobic or toxic terpenoids among the eukaryotic models (Larroude et al., 2018; Banerjee et al., 2024). Similarly, *C. glutamicum* that naturally accumulates carotenoids has emerged as an excellent platform for carotenoid and sesquiterpene production (Li et al., 2021; Stegelmann et al., 2026).

Further, feedstock availability and process sustainability also influence microbial chassis selection. While considering renewable substrates, lignocellulosic hydrolysates, organic acids, and industrial by-products for terpenoid production, microbial hosts, such as *P. putida*, *C. glutamicum*, *B. subtilis* and *Y. lipolytica* can be utilized considering their well known efficiency for these substrates (Phulara et al., 2018a; Sasaki et al., 2019; Li et al., 2022; Carruthers et al., 2025b). Whereas cyanobacteria, which directly convert atmospheric CO_2_ into terpenoids using sunlight, provide an attractive platform for terpenoid biomanufacturing (Pagels et al., 2021; Chenebault et al., 2023; Zhong et al., 2024). They offer a potential platform to reduce dependence on organic carbon feedstocks and improve carbon efficiency; however, the overall sustainability advantage depends on process energy requirements and downstream operations. Here it is also worth noting that although photosynthetic productivity remains lower than heterotrophic systems, recent advances in photosynthetic engineering continue to improve their industrial potential.

Regulatory acceptance is another important consideration, particularly for food, nutraceutical, and therapeutic applications. In this regard, GRAS microbes, such as *B. subtilis*, *C. glutamicum*, *Y. lipolytica*, and *S. cerevisiae* provide advantages owing to their established industrial safety and long history of commercial use. However, the choice among GRAS hosts should also consider substrate preference, product characteristics, and pathway requirements. For example, *B. subtilis* and *C. glutamicum* could be attractive bacterial platforms because of their rapid growth, relatively simple metabolic architecture, and efficient utilization of renewable feedstocks (Phulara et al., 2018a; Sasaki et al., 2019). In contrast, the eukaryotic hosts *S. cerevisiae* and *Y. lipolytica* may be preferred for structurally complex terpenoids requiring cytochrome P450-mediated functionalization (Shukla et al., 2023).

Besides these well-established hosts, several emerging microbial platforms contribute unique advantages. For example, *V. natriegens* enables exceptionally rapid strain development owing to its fast growth rate (Lee et al., 2024), while *Streptomyces* species remain invaluable for the discovery and biosynthesis of structurally diverse natural terpenoids because of their extensive secondary metabolism and powerful post-modification machinery (Hu et al., 2023). In addition to the hosts discussed in this review, several non-model microorganisms, including *Zymomonas mobilis* and *Rhodosporidium toruloides*, have also shown promising potential for terpenoid production owing to their unique metabolic capabilities (Yan et al., 2024; Garcia et al., 2026).

Overall, rational host selection should integrate product chemistry, precursor metabolism, CYP450 compatibility, substrate utilization, product toxicity, downstream processing, regulatory acceptance, and industrial scalability rather than relying solely on reported production titers (Figures 1B,C). Such an application-oriented strategy will facilitate the development of efficient, sustainable, and commercially viable microbial platforms for next-generation terpenoid biomanufacturing.

## 4 Conclusion and future prospects

Microbial terpenoid production has evolved from conventional model organisms to a diverse portfolio of specialized microbial cell factories, each possessing unique physiological and metabolic advantages. Rather than relying on a universal production host, future efforts should focus on application-driven host selection by considering microbial physiology with the product and process complexity and the end use of the target terpenoid. Advances in genome engineering and systems biology integrated with artificial intelligence-guided metabolic engineering, and sustainable feedstock utilization may further enhance terpenoid productivity and process economics. Together with these advances, strategic host selection is expected to accelerate the transition towards green, scalable, and application-specific production of terpenoids for pharmaceuticals, nutraceuticals, flavors, fragrances, and renewable biofuels.
